# Effect of Different Host Plants on Life Type Characteristics of Three Spider Mite Pests (Acari: Prostigmata: Tetranychidae)

**DOI:** 10.3390/ani13223433

**Published:** 2023-11-07

**Authors:** Hafiz Muhammad Saqib Mushtaq, Hafiz Muhammad Sajid Ali, Muhammad Kamran, Fahad Jaber Alatawi

**Affiliations:** Department of Plant Protection, College of Food and Agriculture Sciences, King Saud University, Riyadh 11451, Saudi Arabia; hmushtaq@ksu.edu.sa (H.M.S.M.); hsajid@ksu.edu.sa (H.M.S.A.); murafique@ksu.edu.sa (M.K.)

**Keywords:** citrus brown mite, life types, silken threads, two-spotted spider mite, webbing behavior

## Abstract

**Simple Summary:**

Some spider mite species are economically important agricultural pests, attacking both annual and perennial host plants. They usually produce silken threads of varying densities on the surface of the leaves of inhabiting plants to perform various biological/behavioral activities. In the present study, field-collected leaf samples and laboratory-infested leaves were used to evaluate the effect of different plants on the web-associated behavioral characteristics (life type) of three spider mite species, namely, *Tetranychus urticae*, *Eutetranychus orientalis*, and *Eutetranychus palmatus*. Both annual and perennial plants for *T. urticae* and only perennial plants for *E. orientalis* and *E. palmatus* were used. Two spider mites, *E. orientalis* and *E. palmatus*, showed persistence in life type characteristics on different plant species. In contrast, some behavioral characteristics of *T. urticae* varied by changing the host plants. Although *T. urticae* showed variations in some behavioral characteristics, it did not change its life type, which shows its high adaptability to utilizing the host plant resources. The variations observed in the life type characteristics of *T. urticae* could be helpful in applied pest management.

**Abstract:**

The present study evaluated the host plant effect on life type characteristics of three important spider mite pest species, *Tetranychus urticae* Koch, *Eutetranychus orientalis* (Klein), and *E. palmatus* Attiah (Acari: Prostigmata: Tetranychidae), based on both field and laboratory observations. The polyphagous species, *T. urticae* with complicated web (CW-u) life type, occupying unstable habitats, showed variations in the sites for quiescence (SQ), sites for oviposition (SO), sites for defecation (SD), and webbing density (WD) on different annual/perennial host plants. The SQ, SO, and SD of *T*. *urticae* were observed either on the leaf, web threads, or trichomes. *Tetranychus urticae* constructed the lowest WD on tomato plants and the highest WD on maize/mulberry plants. Two spider mite species of the genus *Eutetranychus* Banks, the polyphagous *E. orientalis* and the oligophagous *E. palmatus*, inhabit stable host plants, depicted in the little web (LW-j) life types with persistency in all characteristics on different plants. It is concluded that polyphagous spider mites have restricted their life types, showing their high adaptability to utilize the resources of different host plants for survival with slight variation in some important life type characteristics.

## 1. Introduction

Spider mites belonging to the family Tetranychidae Donnadieu (Acari: Prostigmata: Tetranychidae) inhabit various annual and perennial host plants [1]. Some members of the subfamily Tetranychinae Berlese are notorious agricultural pests [2,3] and usually produce silken web threads of varying densities and complexities [4,5,6]. Such web structures serve various biological purposes and represent an adaptation for the mite survival on the inhabited host plants [6].

The webbing structures and associated behavioral characteristics of spider mite species on the surface of host plants are technically termed “life types”, and are mainly categorized as little web (LW), complicated web (CW), and woven nest (WN) [4,5]. Further, these three main life types are subdivided into various subtypes based on persistent differences in associated characteristics [4,5]. Among different life type characteristics, the type of the host plant inhabited by the spider mite species is also considered the defining characteristic of a subtype [4].

The idea of classifying spider mites based on their life types was initially proposed over 40 years ago, and numerous studies on the peculiarities of these life types have since been documented [7,8,9]. The life types of different spider mite species belonging to different genera of Tetranychidae have been studied so far [4,5,10,11,12,13,14]. Among them, members of the genera *Tetranychus* Dufour and *Eutetranychus* Banks are severe threats to many different economic host plants [3]. The two spotted spider mite, *Tetranychus urticae* Koch and the citrus brown mite *Eutetranychus orientalis* (Klein), are polyphagous in feeding habits with CW-u and LW-j life types, respectively [4,5]. The oligophagous species, *Eutetranychus palmatus* Attiah, has been reported from two plant families of Arecacea and Malvaceae [14] and is considered a pest of date palm [15]; however, its life type still remains unexplored and needs to be studied.

The “type” of the life type of a spider mite species could depend on its feeding habit (oligophagous or polyphagous) and the type of host plant (annual or perennial) inhabited [4,5,6]. It has been argued that the spider mite species infesting annual host plants (unstable habitat) tend to have a fixed life type and mites infesting perennial host plants (stable habitat) exhibit a different life type [4]. Therefore, this study was designed to assess the life type characteristics of two polyphagous spider mite pests, *T. urticae* and *E*. *orientalis*, feeding on different host plant (annual and perennial) leaves. Also, the life type of the oligophagous pest *E. palmatus* was characterized for the first time.

## 2. Materials and Methods

### 2.1. Collection Sites and Mite Rearing

The populations of *Tetranychus urticae*, *Eutetranychus orientalis*, and *E*. *palmatus* were collected from infested leaves of *Solanum melongena* L., (Solanaceae), *Citrus* sp. (Rutaceae), and *Washingtonia filifera* (Lindl.) (Arecaceae) plants grown within the vicinity of the King Saud University (KSU), Riyadh, Saudi Arabia (SA), respectively, between 2020 and 2021. In addition, a small colony of each spider mite species was reared separately by making leaf arenas of their respective host plants, based on the rearing methods of Mirza et al. [9], with slight modifications. Only the size (3.5–4.5 × 4.5–5.5 cm^2^) and shape (either rectangular or circular) of mites-rearing arenas were modified due to the differences in leaf morphology of respective host plants. All spider mite cultures were kept in a climate-controlled growth chamber (Binder, Tuttlingen, Germany) and maintained at 28 ± 2 °C, 35 ± 10% RH, and L14: D10 photoperiod throughout the experimental duration.

### 2.2. Spider Mites’ Identification

The specimens of each tested spider mite species were mounted on glass slides in Hoyer’s medium under the SZX10 stereomicroscope (Olympus, Tokyo, Japan). In addition, these specimens were taxonomically identified as species using a BX51 fluorescence microscope (Olympus, Tokyo, Japan) with the help of the published taxonomic literature [16,17,18]. Finally, the voucher specimens of each identified/tested species were preserved in the Acarology section of the King Saud University Museum of Arthropods, Riyadh, SA.

### 2.3. Experimental Procedure to Study the Annual and Perennial Host Plants’ Effect on Life Type Characteristics of Polyphagous/Oligophagous Spider Mite Pests in the Laboratory

The experiment was conducted in the Biological Control Laboratory, Department of Plant Protection, KSU, during 2020–2021. The life type characteristics of (a) *T*. *urticae* were examined on six (four annual and two perennial) host plants, *Capsicum annum* L., *S*. *melongena*, *S*. *lycopersicum* L., (Solanaceae), *Morus alba* L. (Moraceae), *Zea mays* L. (Poaceae), and *Ziziphus jujuba* L. (Rhamnaceae); (b) *E*. *orientalis* was examined on three perennial plants, *Citrus* sp., *Ricinus communis* L. (Euphorbiaceae), and *Z*. *jujube*; and (c) *E. palmatus* was examined on two perennial plants, *Phoenix dactylifera* L. and *W*. *filifera* (Arecaceae). Each treatment (=host plant) was replicated 10 times. The life type characteristics of tested spider mite species were studied on the leaf arenas of size (3.5–4.5 × 4.5–5.5 cm^2^) prepared with the leaves of host plant species mentioned above following Mirza et al. [12], in a climate-controlled chamber at (28 ± 2 °C, 35 ± 5% RH, and L14: D10 photoperiod).

In the laboratory experiments, the newly matured females along with conspecific males were released, separately for each of three tested spider mite species, viz., *T. urticae*, *E*. *orientalis*, and *E*. *palmatus*, into their respective experimental arenas. After 3 to 4 days, the mated/gravid females were then used in the experiment. Mites were released near leaf-midrib with the help of a fine camel hairbrush. The life type characteristics of (a) *T*. *urticae* were examined on six (four annual and two perennial) host plants, *Capsicum annum* L., *S*. *melongena*, *S*. *lycopersicum* L., (Solanaceae), *Morus alba* L. (Moraceae), *Zea mays* L. (Poaceae), and *Ziziphus jujuba* L. (Rhamnaceae); (b) *E*. *orientalis* was examined on three perennial plants, *Citrus* sp., *Ricinus communis* L. (Euphorbiaceae), and *Z*. *jujube*; and (c) *E. palmatus* was examined on two perennial plants, *Phoenix dactylifera* L. and *W*. *filifera* (Arecaceae). The experimental arenas were set according to the size (3.5–4.5 × 4.5–5.5 cm^2^) and shape (either rectangular or circular) of each respective plant leaf. Each treatment (=host plant) was replicated 10 times. The experimental arenas were kept in a climate-controlled growth chamber for 10 days and maintained at 28 ± 2 °C, 35 ± 5% RH, and L14: D10 photoperiod. To ensure the establishment of spider mite colonies, all experimental arenas were observed after the 3rd day of mite release under an M165 C Stereomicroscope (LEICA, Wetzlar, Germany). However, the final observational data were recorded on the 10th day of mite release. Due to the biological activities (e.g., feeding and defecation) of spider mites, when time passed, leaf color was slightly changed (green to pale yellow) in some experimental arenas. The leaf side (adaxial or abaxial) of each host plant leaf for each tested spider mite species was selected based on the leaf side of natural infestation. The life type characteristics of *T*. *urticae* and *E*. *orientalis* were evaluated on the adaxial leaf sides of all tested host plants, whereas *E*. *palmatus* was assessed on adaxial and abaxial sides of *P*. *dactylifera* and *W*. *filifera*, respectively.

### 2.4. Observations of Life Type Characteristics on Field-Infested Leaf Samples

The leaf samples of the following mentioned plant species infested naturally with *T*. *urticae*, *E*. *orientalis*, and *E*. *palmatus* were collected from the field and brought to the laboratory. The life type characteristics of the spider mite pest (a) *T*. *urticae* on five (four annual and one perennial) host plants, *C*. *annum*, *S*. *melongena*, *S*. *lycopersicum*, *Z*. *mays*, and *Z*. *jujuba*; (b) *E*. *orientalis* on three perennial plants, *Citrus* sp., *R*. *communis*, and *Z. jujuba*; and (c) *E*. *palmatus* on a perennial host plant, *W*. *filifera*, were observed from these naturally infested leaf samples collected from the field under stereomicroscope in the laboratory, grown within the vicinity of KSU. As compared to the laboratory tests, the life type characteristics of *T. urticae* and *E. palmatus* could not observed on the field-infested leaves of *M. alba* and *P. dactylifera* due to the unavailability of natural infestation, respectively. Randomly, five leaves (=replicates) were collected from each host plant, preserved separately in polyethylene bags, and brought to the Acarology and Biological Control R&D Labs., College of Food and Agriculture Sciences, KSU. Each spider mite species on each host plant leaf was examined under a stereomicroscope to investigate its life type characteristics.

### 2.5. Data Reading and Statistical Analysis

A total of 10 characteristics of spider mite life type were observed, i.e., host plant type (HP; annual, perennial, etc.), leaf side (LS; upper, lower, etc.) inhabited, webbing structure (WS) and density (WD), sites for oviposition (SO), defecation (SD), and quiescence (SQ), spinning during walking (SW), site for feeding and walking (SFW), and egg cover (EC; guy ropes, dense web, etc.) produced by females.

Following Mushtaq et al. [10], the observed life type characteristics for each spider mite species were comparatively investigated on different host plant leaves, and the obtained results were expressed in percentages. Each examined life type characteristic was considered and recorded as 10% and 20% per replicate in the laboratory and field observations, respectively. Moreover, the observational data were separated into supposed ranks (1–6, as in Table S1) for statistical analysis. Additionally, photographs related to observations on some life type characteristics were captured using an Olympus Microscope Camera (DP72) attached to a stereomicroscope. The ranked data (Table S1) were statistically analyzed either through the Kruskal–Wallis test and Wilcoxon two-sample test (Mann–Whitney U-test), and mean scores were ranked by Wilcoxon rank-sums test using the SAS computer program v.9.4 [19].

The webbing density on field-collected and laboratory-infested leaves was quantified by using the methodology adopted by Sabelis [20] and Lemos et al. [21], with slight modifications, i.e., five different webbing density levels: no webbing (0%), low webbing (1–25%), medium webbing (26–50%), high webbing (51–75%), and extremely high webbing (76–100%) were proposed based on differences in obtained WD percentages (Table S1). To quantify the webbing density (%), a white sheet with a 1 cm^2^ hole was placed over the infested leaf. An accurately measured quantity of 3 mg sand was sprinkled through a 1 cm^2^ hole on the web surface. Some of the sand particles were passed through the silken strands of the web and landed on the leaf, while some adhered to the web threads. The webbing density (WD) was calculated by using the following equation.

Webbing density (WD) % = SW/TSP × 100

whereas

SW = Sand particles adhere on/within web threads.

TSP = Total number of sand particles (SW + SL).

SL = Sand particles on the leaf surface, do not adhere to web threads.

## 3. Results

### 3.1. Life Type Characteristics of Tetranychus urticae on Some Annual and Perennial Host Plant Leaves

**Figure 1:**
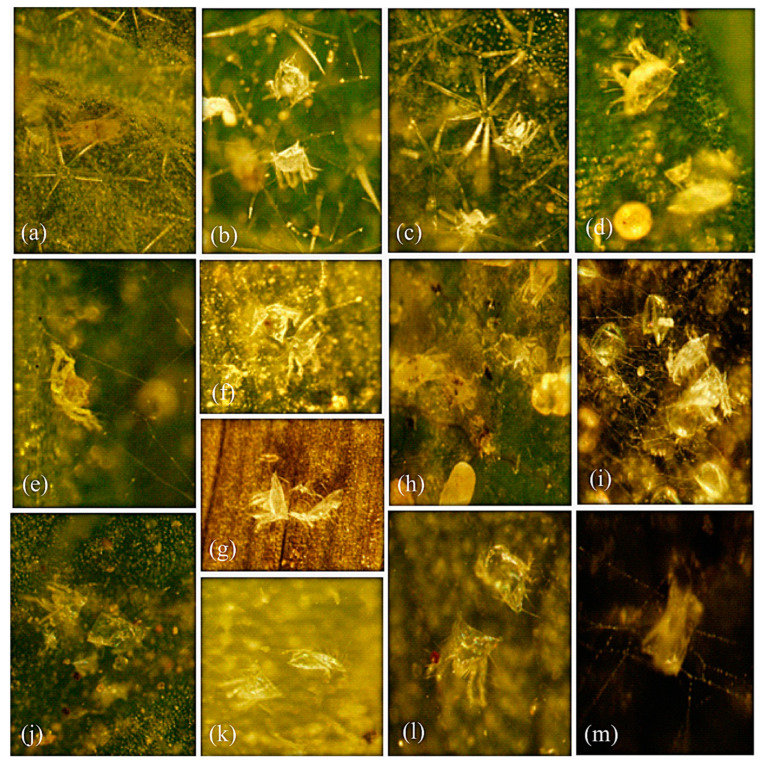
Variations were observed in site for quiescence (SQ) shown by *Tetranychus urticae* on the adaxial leaf sides of different (annual and perennial) host plant leaves (*Solanum melongena*, *Capsicum annum*, *Zea mays*, *Morus alba*, *S. lycopersicum*, and *Ziziphus jujuba*) in the laboratory and/or field observations. *S. melongena*: SQ on (**a**) leaf, (**b**) web, and (**c**) trichome; *C. annum*: on (**d**) leaf and (**e**) web; *Z. mays*: on (**f**) leaf and (**g**) web; *M. alba:* on (**h**) leaf and (**i**) web; *S. lycopersicum*: on (**j**) leaf and (**k**) web; and *Z. jujuba*: on (**l**) leaf and (**m**) web.

**Figure 2:**
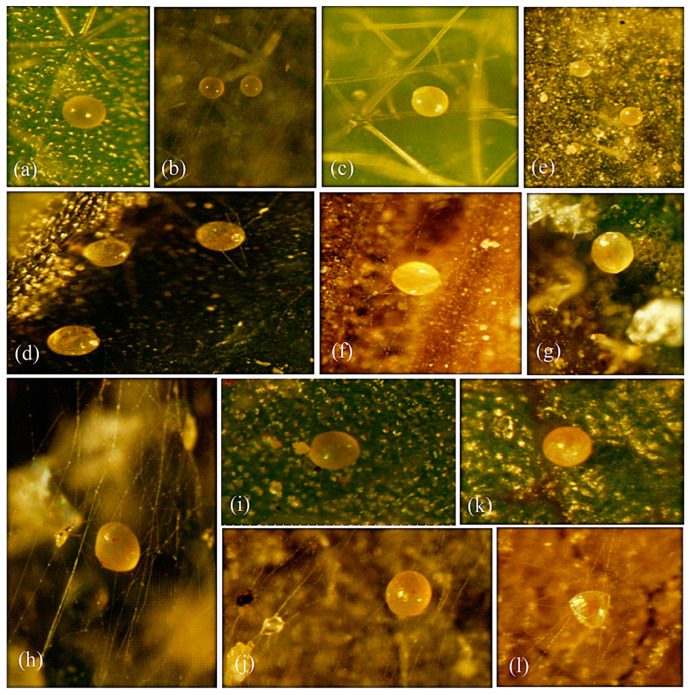
Variations were observed in the site for oviposition (SO) exhibited by *Tetranychus urticae* on the adaxial leaf sides of different (annual and perennial) host plant leaves (*Solanum melongena*, *Capsicum annum*, *Zea mays*, *Morus alba*, *S. lycopersicum*, and *Ziziphus jujuba*) in the laboratory and field observations. *S. melongena*: SO on (**a**) leaf, (**b**) web, and (**c**) trichome; *C. annum*: on (**d**) leaf and web; *Z. mays*: on (**e**) leaf and (**f**) web; *M. alba:* on (**g**) leaf and (**h**) web; *S. lycopersicum*: on (**i**) leaf and (**j**) web; and *Z. jujuba*: on (**k**) leaf and (**l**) web.

**Figure 3:**
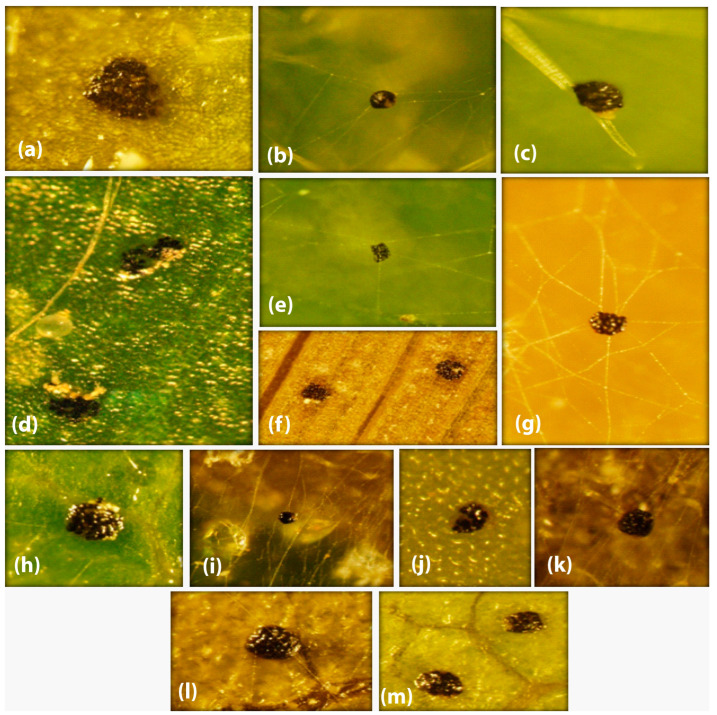
Variations were observed in the site for defecation (SD) shown by *Tetranychus urticae* on the adaxial leaf sides of different (annual and perennial) host plant leaves (*Solanum melongena*, *Capsicum annum*, *Zea mays*, *Morus alba*, *S. lycopersicum*, and *Ziziphus jujuba*) in the laboratory and field observations. *S. melongena*: SD on (**a**) leaf, (**b**) web, and (**c**) trichome; *C. annum*: on (**d**) leaf and (**e**) web; *Z. mays*: on (**f**) leaf and (**g**) web; *M. alba:* on (**h**) leaf and (**i**) web; *S. lycopersicum*: on (**j**) leaf and (**k**) web; and *Z. jujuba*: on (**l**) leaf and (**m**) web.

The results confirmed that the polyphagous spider mite species *T. urticae* did not change its life type (CW-u) either on four annual (unstable habitat) or two perennial (stable habitat) host plant leaves. Some behavioral characteristics, i.e., WS, EC, SFW, and SW, of *T. urticae* remained persistent on tested annual/perennial plant leaves in laboratory experiments and on field-collected leaves samples (Table 1 and Table 2). However, the SQ, SO, SD, and WD were found to be variable (Table 1 and Table 2; Figure 1, Figure 2 and Figure 3). In laboratory experiments, four life type characteristics, i.e., SQ (H = 38.268), SO (H = 49.820), SD (H = 56.420), and WD (H = 39.826) (all df = 5, *p* < 0.05) of *T. urticae* showed significant differences among six tested host plant leaves (Table 3). The SQ was significantly different in *S. melongena* (vs. *Z. mays*, *M. alba*, *S. lycopersicum*), *C. annum* (vs. *Z. mays*, *M. alba*, *S. lycopersicum*), *Z. mays* (vs. *Z. jujuba*), and *M. alba* (vs. *Z. jujuba*) (Table 3). The SO was significantly different in *S. melongena* (vs. *C. annum*, *Z. mays*, *M. alba*, *S. lycopersicum*, *Z. jujuba*), *C. annum* (vs. Z. mays, *S. lycopersicum*, *Z. jujuba*; Table), *Z. mays* (vs. M. alba), and *M. alba* (vs. *S. lycopersicum*, *Z. jujuba*) (Table 3). The SD was significantly different in *S. melongena* (vs. *C. annum*, *Z. mays*, *M. alba*, *S. lycopersicum*, *Z. jujuba*) and *C. annum* (vs. *Z. mays*, *M. alba*, *S. lycopersicum*, *Z. jujuba* (Table 3). The WD was significantly different in *S. melongena* (vs. *C. annum*, *S. lycopersicum*), *C. annum* (vs. *Z. mays*, *M. alba*, *S. lycopersicum*, *Z. jujuba*; Table 3), *Z. mays* (vs. *S. lycopersicum*), *M. alba* (vs. *S. lycopersicum*), and *S. lycopersicum* (vs. *Z. jujuba*) (Table 3).

Similarly, the field observations also showed significant differences in SQ (H = 23.073), SO (H = 23.073), SD (H = 23.073), and WD (H = 20.947) (all df = 4, *p* < 0.05) of *T. urticae* among five (four annual and one perennial) tested host plants (Table 4). The SQ, SO, and SD were significantly different in *S. melongena* (vs. *C. annum*, *Z*. *mays*, *S. lycopersicum*, *Z. jujuba*), *C. annum* (vs. *Z*. *mays*, *S. lycopersicum*, *Z. jujuba*), and *Z. mays* (vs. *S. lycopersicum*, *Z. jujuba*) (Table 4), whereas the WD was significantly different in *S. melongena* (vs. *C. annum*, *Z*. *mays*, *S. lycopersicum*), *C. annum* (vs. *S. lycopersicum*, *Z. jujuba*), *Z. mays* (vs. *S. lycopersicum*, *Z. jujuba*), and *S. lycopersicum* (vs. *Z. jujuba*) (Table 4).

### 3.2. Life Type Characteristics of Eutetranychus orientalis

The results revealed that life type behavioral characteristics of polyphagous *E. orientalis* remained persistent on tested perennial plants, both in the laboratory and in field observations (Table 1 and Table 2). The life type of *E. orientalis* also did not change on different perennial plants.

### 3.3. Life Type Characteristics of Eutetranychus palmatus

The life type characteristics of *E. palmatus* were investigated for the first time in the present study, and its life type also remained the same on two perennial host plants. It was observed that all the behavioral characteristics of *E. palmatus* remained persistent both in the laboratory and in field observations (Table 1 and Table 2).

**Figure 4:**
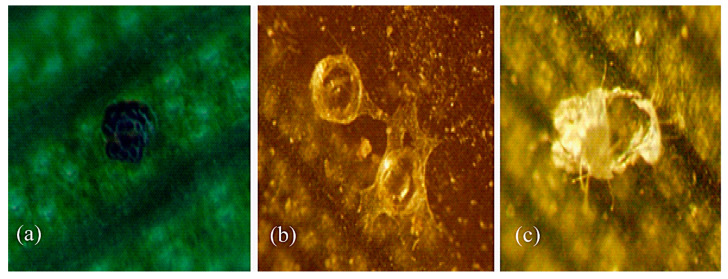
Some important life type (LW-j) characteristics of *Eutetranychus palmatus* on the adaxial and abaxial leaf sides of *P. dactylifera* and *W. filifera* (perennial) were observed in field and laboratory observations. (**a**) Feces on the leaf surface; (**b**) egg covers as dense web; (**c**) quiescence stage on the leaf surface.

In the laboratory and field observations, mobile stages of *E. palmatus* neither spun a web while walking on the leaf surface nor showed dragging behavior. The quiescent stages/exuviae, eggs, and feces were consistently observed on the leaf surface (Figure 4), near to or away from the leaf midrib. The female of *E. palmatus* constructed egg covers as dense webs (Figure 4b) and showed weaving behavior. Females, males, nymphs, and larvae were randomly observed during feeding, walking, and resting on the leaf surface near to or away from the midrib. *Eutetranychus palmatus* showed an LW life type and LW-j subtype based on the observed behavioral characteristics on the leaves of *P. dactylifera* and *W. filifera*.

## 4. Discussion

In the present study, the host plant effect was assessed on the life type characteristics of three spider mite pests, *T. urticae*, *E. orientalis*, and *E. palmatus*. The four life type characteristics, i.e., site for quiescence (SQ), site for oviposition (SO), site for defecation (SD), and webbing density (WD) of *T. urticae* were observed either on the leaf surface, web threads, or trichomes and varied within and between different host plants (annual and perennial) (Table 1 and Table 2, Figure 1, Figure 2 and Figure 3). The life type characteristics of *T. urticae* were not previously studied regarding the annual type of host plants. However, the CW-u life type was determined for *T. urticae* on *Sambucus sieboldiana* (perennial), where the preferred SQ, SO, and SD were on threads of an irregularly complicated web [4]. Such variations in the life type characteristics could be due to the change in the microhabitat, leaf structure (glabrous or pubescence), and mite population density [4,10,22].

*Tetranychus urticae* is a polyphagous pest with >1100 annual and perennial hosts [14,15]. Although Saito [4] indicated that polyphagous mite species inhabiting annual and perennial host plants exhibit fixed and diverse life type, respectively. In contrast, *T. urticae* showed significant variations in the SQ, SO, SD, and WD on different annual and perennial plant leaves in the present study (Table 1 and Table 2, Figure 1, Figure 2 and Figure 3). Similarly, an oligophagous species, *Oligonychus afrasiaticus* (McGregor) having CW-d life type, showed variations in SO and SQ when tested on annual (*Sorghum bicolor* (Poaceae)) and perennial (*Saccharum officinarum* L. (Poaceae)) host plants without changing their life type [13]. It has been reported that some polyphagous spider mite pests change their life type on perennial plants [4,11,22]. For example, *Eotetranychus tilliarium* (Hermann) showed the CW-r life type on the hairy leaves of *Alnus hirsuta* Turcaz. (Betulaceae), and the WN-t life type on glabrous leaves of *A*. *japonica* Steud [4]. Likewise, *E*. *asiaticus* Ehara changed its life type characteristics on two different perennial plants by depicting the WN-t life type on strawberry leaves [23], and the CW-g life type when inhabiting the galls created by an insect species, *Trioza cinnamomi* (Boselli) (Hemiptera: Triozidae) on leaves of *Cinnamomum japonicum* Siebold (Laureacea) [11].

The amount of webbing (WD, an essential characteristic of CW-life type) produced by *T. urticae* was reported to be affected due to changes in environmental conditions, host plants inhabited, and mite population density [23,24,25]. In the present study, the WD of *T. urticae* varied on annual host plants from low (on tomato) to extremely high (e.g., on brinjal) (Table 1 and Table 2). On the other hand, the WD range was high to extremely high on the tested perennial plants. Similarly, *T. urticae* showed variations in the amount of web deposition when tested on seven perennial host plants, i.e., Algerian ivy, bean, cotton, castor bean, hibiscus, rose, and sweet potato [26,27]. These variations in the WD of *T. urticae* are probably due to the differences in the physical structure of the leaves of tested host plants. Because leaf depressions (e.g., along leaf midribs) play a crucial role as the basis for the construction of complicated web structures, females of *T. urticae* showed aggregation behavior near such depressions [6].

In the present study, two congeneric spider mite pests, the polyphagous *E. orientalis* (>200 hosts) and the oligophagous *E. palmatus* (six hosts) [14], did not show variations/differences in any life type characteristics on different perennial host plants (Table 1 and Table 2). The life type of *E. palmatus* (LW-j) was investigated for the first time in this study. Previously, the LW-j life type was also detected for *E. orientalis* on the leaves of a perennial plant, *Manihot glaziovii* Müll. Arg. (Euphorbiaceae) [10]. In the present study, these two *Eutetranychus* species showed persistency in life type characteristics on different perennial host plant leaves. It could be due to the fact that LW is the primary and simplest life type [4,6], and is observed in less advanced Tetranychinae genera, e.g., *Aponychus* Rimando, *Eurytetranychus* Oudemans, *Eutetranychus* Banks, *Panonychus* Yokoyama, *Stylophoronychus* Prasad, and *Yezonychus* Ehara [4]. The members (e.g., *E. orientalis* and *E. palmatus*) of the tribe Eurytetranychini Reck are considered more primitive than the members (e.g., *T. urticae* and *Oligonychus* spp.) of the tribe Tetranychini Reck [6]. It could be one of the reasons why, in the LW-j life type of *E. orientalis* and *E. palmatus*, pest species never spin threads as dense webs while walking on the leaf surface (dragging behavior), but females produce dense web covers on eggs (weaving behavior) to protect their progeny [28,29,30,31,32].

## 5. Conclusions

It is concluded that the tested spider mites have restricted life types with variations in some life type characteristics, which shows their high adaptability to utilize the host plant resources. The variations observed in the life type characteristics of *T. urticae* could be helpful in applied pest management (e.g., in the proper selection of potential biological control agents) when infesting various economic plants.

## Figures and Tables

**Table 1:** Differences and variations (estimated in %) were observed in some important life type characteristics of *Tetranychus urticae*, *Eutetranychus orientalis*, and *E. palmatus* on adaxial or abaxial leaf sides of different host plants under laboratory conditions.

Spider Mite Species	Host Plants	Host Plant Type (HP)	No. of Obs. (N)	Life Type Characteristics																						
				Site for Quiescence (SQ) %						Site for Oviposition (SO) %						Site for Defecation (SD) %						Webbing Density (WD) %				
				W	L	WL	WT	T	WTL	W	L	WL	WT	T	WTL	W	L	WL	WT	T	WTL	NW	LW	MW	HW	EHW
*T. urticae*	*Solanum melongena*	Annual	10	0	100	0	0	0	0	0	0	0	0	0	100	0	0	0	0	0	100	0	0	0	40	60
	*Capsicum annum*	Annual	10	0	100	0	0	0	0	0	100	0	0	0	0	0	100	0	0	0	0	0	10	80	10	0
	*Zea mays*	Annual	10	0	0	100	0	0	0	0	0	100	0	0	0	0	0	100	0	0	0	0	0	10	20	70
	*Morus alba*	Perennial	10	0	0	100	0	0	0	0	70	30	0	0	0	0	0	100	0	0	0	0	0	10	20	70
	*S. lycopersicum*	Annual	10	0	20	80	0	0	0	0	20	80	0	0	0	0	10	90	0	0	0	0	100	0	0	0
	*Ziziphus jujuba*	Perennial	10	30	20	50	0	0	0	0	0	100	0	0	0	0	0	100	0	0	0	0	10	10	40	40
*E. orientalis*	*Ricinus communis*	Perennial	10	0	100	0	0	0	0	0	100	0	0	0	0	0	100	0	0	0	0	100	0	0	0	0
	*Citrus* sp.	Perennial	10	0	100	0	0	0	0	0	100	0	0	0	0	0	100	0	0	0	0	100	0	0	0	0
	*Z. jujuba*	Perennial	10	0	100	0	0	0	0	0	100	0	0	0	0	0	100	0	0	0	0	100	0	0	0	0
*E. palmatus*	*Phoenix dactylifera*	Perennial	10	0	100	0	0	0	0	0	100	0	0	0	0	0	100	0	0	0	0	100	0	0	0	0
	*Washingtonia filifera*	Perennial	10	0	100	0	0	0	0	0	100	0	0	0	0	0	100	0	0	0	0	100	0	0	0	0

W = on/within web threads, L = on leaf surface, WL = on/within web threads and on leaf surface, WT = on/within web threads and on trichrome, T = on trichrome, WTL = on/within web threads, on trichrome and on leaf surface, NW = no webbing, LW = low webbing, MW = moderate webbing, HW = high webbing, EHW = extremely high webbing.

**Table 2:** Differences and variations (estimated in %) were observed in some important life type characteristics of *Tetranychus urticae*, *Eutetranychus orientalis*, and *E. palmatus* on adaxial or abaxial sides of field-infested leaves of different host plants.

Spider Mite Species	Host Plants	Host Plant Type (HP)	No. of Obs. (N)	Life Type Characteristics																						
				Site for Quiescence (SQ) %						Site for Oviposition (SO) %						Site for Defecation (SD) %						Webbing Density (WD) %				
				W	L	WL	WT	T	WTL	W	L	WL	WT	T	WTL	W	L	WL	WT	T	WTL	NW	LW	MW	HW	EHW
*T. urticae*	*Solanum melongena*	Annual	5	0	0	0	0	0	100	0	0	0	0	0	100	0	0	0	100	0	0	0	0	0	0	100
	*Capsicum annum*	Annual	5	100	0	0	0	0	0	100	0	0	0	0	0	100	0	0	0	0	0	0	0	40	60	0
	*Zea mays*	Annual	5	0	80	20	0	0	0	0	80	20	0	0	0	0	80	20	0	0	0	0	0	60	40	0
	*S. lycopersicum*	Annual	5	0	0	100	0	0	0	0	0	100	0	0	0	0	0	100	0	0	0	0	100	0	0	0
	*Ziziphus jujuba*	Perennial	5	0	0	100	0	0	0	0	0	100	0	0	0	0	0	100	0	0	0	0	0	0	40	60
*E. orientalis*	*Ricinus communis*	Perennial	5	0	100	0	0	0	0	0	100	0	0	0	0	0	100	0	0	0	0	100	0	0	0	0
	*Citrus* sp.	Perennial	5	0	100	0	0	0	0	0	100	0	0	0	0	0	100	0	0	0	0	100	0	0	0	0
	*Z. jujuba*	Perennial	5	0	100	0	0	0	0	0	100	0	0	0	0	0	100	0	0	0	0	100	0	0	0	0
*E. palmatus*	*Washingtonia filifera*	Perennial	5	0	100	0	0	0	0	0	100	0	0	0	0	0	100	0	0	0	0	100	0	0	0	0

W = on/within web threads, L = on leaf surface, WL = on/within web threads and on leaf surface, WT = on/within web threads and on trichrome, T = on trichrome, WTL = on/within web threads, on trichrome and Pl on leaf surface, NW = no webbing, LW = low webbing, MW = moderate webbing, HW = high webbing, EHW = extremely high webbing.

**Table 3:** Wilcoxon two-sample test results of host plants’ effect on some important life type characteristics (SQ, SO, SD, and WD) of *Tetranychus urticae* on adaxial leaf sides of different host plants in the laboratory.

Host Plants Comparison	Site for Quiescence (SQ)			Site for Defecation (SO)			Site for Oviposition (SD)			Webbing Density (WD)		
	*U*	DF	*p*	*U*	DF	*p*	*U*	DF	*p*	*U*	DF	*p*
*Solanum melongena* vs. *Capsicum annum*	0.000	1	1.0000	19.000	1	<0.0001	19.000	1	<0.0001	14.702	1	0.0001
*S. melongena* vs. *Zea mays*	19.000	1	<0.0001	19.000	1	<0.0001	19.000	1	<0.0001	0.074	1	0.7864
*S. melongena* vs. *Morus alba*	19.000	1	<0.0001	17.195	1	<0.0001	19.000	1	<0.0001	0.074	1	0.7864
*S. melongena* vs. *S. lycopersicum*	12.667	1	0.0004	17.593	1	<0.0001	18.182	1	<0.0001	16.964	1	<0.0001
*S. melongena* vs. *Zizipus jujuba*	0.745	1	0.3880	19.000	1	<0.0001	19.000	1	<0.0001	1.378	1	0.2404
*C. annum* vs. *Z. mays*	19.000	1	<0.0001	19.000	1	<0.0001	19.000	1	<0.0001	12.794	1	0.0003
*C. annum* vs. *M. alba*	19.000	1	<0.0001	3.353	1	0.0671	19.000	1	<0.0001	12.794	1	0.0003
*C. annum* vs. *S. lycopersicum*	12.667	1	0.0004	12.667	1	0.0004	15.546	1	<0.0001	15.000	1	0.0001
*C. annum* vs. *Z. jujuba*	0.745	1	0.3880	19.000	1	<0.0001	19.000	1	<0.0001	7.234	1	0.0072
*Z. may* vs. *M. alba*	0.000	1	1.0000	10.231	1	0.0014	0.000	1	1.0000	0.000	1	1.0000
*Z. may* vs. *S. lycopersicum*	2.111	1	0.1462	2.111	1	0.1462	1.000	1	0.3173	17.148	1	<0.0001
*Z. may* vs. *Z. jujuba*	6.209	1	0.0127	0.000	1	1.0000	0.000	1	1.0000	1.700	1	0.1923
*M. alba* vs. *S. lycopersicum*	0.146	1	2.1111	4.798	1	0.0285	1.000	1	0.3173	17.148	1	<0.0001
*M. alba* vs. *Z. jujuba*	6.209	1	0.0127	10.231	1	0.0014	0.000	1	1.0000	1.700	1	0.1923
*S. lycopersicum* vs. *Z. jujuba*	2.587	1	0.1078	2.111	1	0.1462	1.000	1	0.3173	14.119	1	0.0002

**Table 4:** Wilcoxon two-sample test results of host plants’ effect on some important life type characteristics (SQ, SO, SD, and WD) of *Tetranychus urticae* on adaxial leaf sides of field-infested leaves of different host plants.

Host Plants Comparison	Site for Quiescence (SQ)			Site for Oviposition (SO)			Site for Defecation (SD)			Webbing Density (WD)		
	*U*	DF	*p*	*U*	DF	*p*	*U*	DF	*p*	*U*	DF	*p*
*Solanum melongena* vs. *Capsicum annum*	9.000	1	0.003	9.000	1	0.003	9.000	1	0.003	8.036	1	0.005
*S. melongena* vs. *Zea mays*	8.333	1	0.004	8.333	1	0.004	8.333	1	0.004	8.036	1	0.005
*S. melongena* vs. *S. lycopersicum*	9.000	1	0.003	9.000	1	0.003	9.000	1	0.003	9.000	1	0.003
*S. melongena* vs. *Zizipus jujuba*	9.000	1	0.003	9.000	1	0.003	9.000	1	0.003	2.250	1	0.134
*C. annum* vs. *Z. mays*	8.333	1	0.004	8.333	1	0.004	8.333	1	0.004	0.360	1	0.549
*C. annum* vs. *S. lycopersicum*	9.000	1	0.003	9.000	1	0.003	9.000	1	0.003	8.036	1	0.005
*C. annum* vs. *Z. jujuba*	9.000	1	0.003	9.000	1	0.003	9.000	1	0.003	4.641	1	0.031
*Z. may* vs. *S. lycopersicum*	6.000	1	0.014	6.000	1	0.014	6.000	1	0.014	8.036	1	0.005
*Z. may* vs. *Z. jujuba*	6.000	1	0.014	6.000	1	0.014	6.000	1	0.014	5.400	1	0.020
*S. lycopersicum* vs. *Z. jujuba*	0.000	1	1.000	0.000	1	1.000	0.000	1	1.000	8.036	1	0.005

## Data Availability

All necessary data for the manuscript is provided.

## Supplementary Materials

The following supporting information can be downloaded at: https://www.mdpi.com/article/10.3390/ani13223433/s1, Table S1: Coding for statistical analysis of laboratory and field observations of life type characteristics.
