# Supplementary material for: Effect of Different Host Plants on Life Type Characteristics of Three Spider Mite Pests (Acari: Prostigmata: Tetranychidae)
Source: Animals (Basel). 2023 Nov 7;13(22):3433. doi: 10.3390/ani13223433 (PMC10668636; doi:10.3390/ani13223433)
Supplement: Supplementary file 1 [file animals-13-03433-s001.zip › animals-2672994-SI.pdf]

Effect of Different Host Plants on Life Type Characteristics of Three Spider Mite Pests (Acari: Prostigmata: Tetranychidae)

Hafiz Muhammad Saqib Mushtaq, Hafiz Muhammad Sajid Ali, Muhammad Kamran and Fahad Jaber Alatawi *

Department of Plant Protection, College of Food and Agriculture Sciences, King Saud University, P.O. Box 2460, Riyadh 11451, Saudi Arabia; hmushtaq@ksu.edu.sa (H.M.S.M.); hsajid@ksu.edu.sa (H.M.S.A.); murafique@ksu.edu.sa (M.K.)

* Correspondence: falatawi@ksu.edu.sa

No. of supplementary tables: 1

Table S1 Coding for statistical analysis of laboratory and field observations of life type characteristics

**Coding for tested spider mite species**						
Tetranychus urticae	1					
Eutetranychus orientalis	2					
Eutetranychus palmatus	3					
**Coding for tested host plants with *T. urticae***						
T1 (*Solanum melongena*)	1					
T2 (*Capsicum annum*)	2					
T3 (*Zea mays*)	3					
T4 (*Morus alba*)	4					
T5 (*Solanum lycopersicum*)	5					
T6 (*Ziziphus jujuba*)	6					
**Coding for tested host plants with *E. orientalis***						
T1 (*Ricinus communis*)	1					
T2 (*Citrus* sp.)	2					
T3 (*Ziziphus jujuba*)	3					
**Coding for tested host plants with *E. palmatus***						
T1 (*Phoenix dactylifera*)	1					
T2 (*Washingtonia filifera*)	2					
**Coding for life type characteristics**						
Host plant type **(HP)**	**1** (Broad-leaved deciduous)	**2** (Broad-leaved evergreen)	**3** (Narrow-leaved deciduous)	**4** (Narrow-leaved evergreen)	**5** (Broad leaved annual)	**6** (Narrow leaved annual)
Leaf side inhabited **(LS)**	**1** (Upper)	**2** (Under)	**3** (Both)			
Webbing structure **(WS)**	**1** (Little web, LW)	**2** (Complicated web, **CW**)	**3** (Woven nest, **WN**)			
Site for quiescence **(SQ)**	**1** (On/within web threads)	**2** (On leaf surface)	**3** (Both on/within web threads & on leaf surface)	**4** (Both on/within web threads & trichrome)	**5** (On trichrome)	**6** (Both on/within web, trichrome & leaf)
Site for oviposition **(SO)**	**1** (On/within web threads)	**2** (On leaf surface)	**3** (Both on/within web threads & on leaf surface)	**4** (Both on/within web threads & trichrome)	**5** (On trichrome)	**6** (Both on/within web, trichrome & leaf)
Site for defecation **(SD)**	**1** (On/within web threads)	**2** (On leaf surface)	**3** (Both on/within web threads & on leaf surface)	**4** (Both on/within web threads & trichrome)	**5** (On trichrome)	**6** (Both on/within web, trichrome & leaf)
Egg covers produced **(EC)**	**1** (Egg covers present)	**2** (Egg covers absent)	**3** (some eggs with/without web covers)			
Site for feeding & walking **(SFW)**	**1** (On, in & under the web)	**2** (Openly away from the web)	**3** (Both on, in and under & openly away from the web)			
Spinning during walking **(SW)**	**1** (without)	**2** (with)				
Webbing density **(WD)**	**1** (0%; no)	**2** (1-25%; low)	**3** (26-50%; medium)	**4** (51-75%; high)	**5** (76-100%; extremely high)	
